# Image analysis and teaching strategy optimization of folk dance training based on the deep neural network

**DOI:** 10.1038/s41598-024-61134-y

**Published:** 2024-05-13

**Authors:** Zhou Li

**Affiliations:** 1 Art College of Shaanxi University of Technology, Hanzhong, 723001 Shaanxi China; 2 Universidad Católica San Antonio de Murcia, 30335 Murcia Region, Spain

**Keywords:** Deep neural network, Folk dance training, Image analysis, Teaching strategy, Classification model, Target detection model, Mathematics and computing, Computer science, Information technology

## Abstract

To improve the recognition effect of the folk dance image recognition model and put forward new suggestions for teachers’ teaching strategies, this study introduces a Deep Neural Network (DNN) to optimize the folk dance training image recognition model. Moreover, a corresponding teaching strategy optimization scheme is proposed according to the experimental results. Firstly, the image preprocessing and feature extraction of DNN are optimized. Secondly, classification and target detection models are established to analyze the folk dance training images, and the C-dance dataset is used for experiments. Finally, the results are compared with those of the Naive Bayes classifier, K-nearest neighbor, decision tree classifier, support vector machine, and logistic regression models. The results of this study provide new suggestions for teaching strategies. The research results indicate that the optimized classification model shows a significant improvement in classification accuracy across various aspects such as action complexity, dance types, movement speed, dance styles, body dynamics, and rhythm. The accuracy, precision, recall, and F1 scores have increased by approximately 14.7, 11.8, 13.2, and 17.4%, respectively. In the study of factors such as different training images, changes in perspective, lighting conditions, and noise interference, the optimized model demonstrates a substantial enhancement in recognition accuracy and robustness. These findings suggest that, compared to traditional models, the optimized model performs better in identifying various dances and movements, enhancing the accuracy and stability of classification. Based on the experimental results, strategies for optimizing the real-time feedback and assessment mechanism in folk dance teaching, as well as the design of personalized learning paths, are proposed. Therefore, this study holds the potential to be applied in the field of folk dance, promoting the development and innovation of folk dance education.

## Introduction

In the context of the rapid development of contemporary educational technology, as a vital part of traditional culture, the teaching and inheritance methods of folk dance need to be innovated and improved^1^. With the rapid advancement of artificial intelligence technology, particularly in deep learning (DL) and image recognition, new perspectives and methods have been provided for folk dance education. Despite the successful applications of these technologies in various fields, their use in folk dance instruction is still in its early stages and faces multiple challenges, such as the accuracy of motion recognition and the personalized design of teaching strategies^2,3^. The motivation of this study is to achieve precise identification and classification of dance movements through the optimization of image recognition models and teaching strategies. This aims to provide real-time feedback for teachers and learners, thereby enhancing the effectiveness of teaching and learning. As a part of cultural heritage, folk dance preservation and dissemination confront challenges in the rapidly changing landscape of modern society. Utilizing the latest technology to optimize teaching strategies contributes to better safeguarding and passing on these precious cultural resources.

This study aims to employ Deep Neural Network (DNN) technology for the analysis of images in folk dance training and proposes corresponding methods for optimizing teaching strategies. The use of DNN technology can enhance the effectiveness and quality of folk dance training, enabling learners to better master dance techniques. Initially, the optimization of image preprocessing and feature extraction in DNN is addressed in this study. Subsequently, the analysis of folk dance training images is conducted using classification and target detection models. Finally, the effectiveness of the model is validated through experiments. Through the exploration, it is hoped that the potential of DNN in folk dance training can be explored, offering an innovative approach to enhance dancers’ technical proficiency and enrich the artistic expression of folk dance.

## Literature review

In traditional studies, Shailesh and Judy (2020) used DL technology for human movement recognition in dance performances. They employed convolutional and recurrent neural networks to extract and analyze the dancers’ posture data. By training the model, different dance movements can be accurately identified, and feedback and guidance can be provided to the dancers. The research results denoted that DL technology had high accuracy and effect in dance training^4^. Yadav et al.^5^ underscored the potential of DL technology in recognizing and tracking hand movements in complex backgrounds, providing crucial technical references and insights for this study. Yadav et al.^6^ discussed the application of gesture recognition technology in human–computer interaction (HCI). By developing a gesture recognition system for a virtual keyboard interface, the study demonstrated the high efficiency of DL models in accurately tracking and identifying gestures. Sarma et al.^7^ further expanded the application of DL in the field of gesture recognition. They adopted an attention-based approach to improving the accuracy of hand semantic segmentation and gesture recognition, offering a valuable methodology for discerning subtle differences in complex dance movements. Yadav et al.^8^ proposed an end-to-end hand positioning system, providing a comprehensive analysis and solution for HCI. The development of this system showcased the effectiveness and flexibility of DL technology in handling complex interactive tasks.

From previous research, it is evident that while these studies have made progress in gesture recognition and HCI, they primarily focus on the recognition of generic gestures or simple movements. There is a lack of in-depth exploration of the identification and analysis of complex folk dance movements and details. Additionally, existing studies often perform well on specific datasets, but the generalization capability and robustness of models still need improvement when faced with complex backgrounds, varying lighting conditions, or diverse dance styles. This study concentrates on the characteristics of folk dance training images, optimizing the image preprocessing and feature extraction steps of DNNs to better adapt to the recognition of intricate dance movements and details. By introducing more complex datasets and testing under different conditions, the model proposed in this study exhibits remarkable improvements in generalization capability and robustness compared to traditional models.

## The folk dance training image model based on DNN

### Application of the folk dance training image model in DNN

Traditional folk dance image recognition methods have some areas for improvement. First, the feature representation is limited. Traditional methods usually use hand-designed feature representation methods, such as color, texture, and shape^9–11^. However, these methods often fail to adequately capture the complexity and diversity of dance movements, limiting the expressive power and performance of recognition models. Second, it is difficult to make subjective judgments and standardization. Traditional methods’ recognition and correction of dance movements often rely on teachers’ subjective judgment and experience^12^. Because of the subjective and artistic nature of dance movements, it is difficult to establish a unified standardized index and evaluation system, which leads to the inaccuracy and lack of objectivity of teaching strategies. Lastly, information extraction is not comprehensive, and traditional methods mainly focus on the external movement characteristics of dancers^13–16^. Nevertheless, folk dance also contains rich information such as cultural background, emotional expression, and artistic style, which are of great significance to the dancers’ recognition and understanding, but are often overlooked. To overcome the deficiencies of traditional folk dance image recognition methods, this study introduced DNN to optimize the recognition model and proposed a corresponding teaching strategy optimization scheme based on experimental results^17^. By adopting DL technology, the characteristics of dance movements can be captured more accurately, and more personalized and effective teaching support can be provided. The application of DNN in folk dance image recognition has shown many advantages, as outlined in Table 1.

**Table 1:** Advantages of DNN in folk dance image recognition.

Advantage	Concrete content
Feature learning and representation learning	High-level abstract feature representations can be learned from the original image data through multi-layer nonlinear transformations. This feature learning ability enables the network to automatically extract and represent richer and more informative image features, which helps improve the accuracy of recognizing dance movements
Data-driven capability	Deep neural networks are data-driven methods that can be trained through large-scale datasets and learn statistical patterns of dance movements
Classification and recognition ability	Deep neural networks are widely used in image classification and target recognition tasks, and their excellent classification ability is also reflected in folk dance image recognition
Interpretability and visualization	Teachers’ understanding and guidance on teaching strategies are enhanced by visually displaying the extraction and expression of dance features through the network through visualization technology

The target detection model is an algorithm that can accurately locate and identify multiple image targets. The application of this model in dance image recognition is crucial to locate and recognize dance movements accurately^18,19^. It is mainly reflected in the following aspects, as exhibited in Table 2.

**Table 2:** Importance of the target detection model in dance image recognition.

Importance	Concrete content
Positioning dance movements	The target detection model can provide more detailed and accurate information by locating specific actions in dance images, facilitating understanding and analysis of dance actions
Multi-target recognition	Dance images often involve multiple dancers performing different movements simultaneously, which requires independent recognition and analysis of each dancer’s movements
Attitude estimation and action sequence	Dance movements involve complex postures and sequences of movements. The target detection model and human pose estimation algorithms can accurately identify and track dancers’ key points and joints

The target detection model plays a vital role in dance image recognition. It can realize the accurate positioning and recognition of dance movements, multi-objective processing, individual movement recognition, pose estimation, and movement sequence analysis, thus providing more in-depth and comprehensive dance teaching support. By combining with DNN, the target detection model can further improve the effect and accuracy of the dance image recognition model^20–22^.

### Image preprocessing and feature extraction optimization

**Figure 1:**
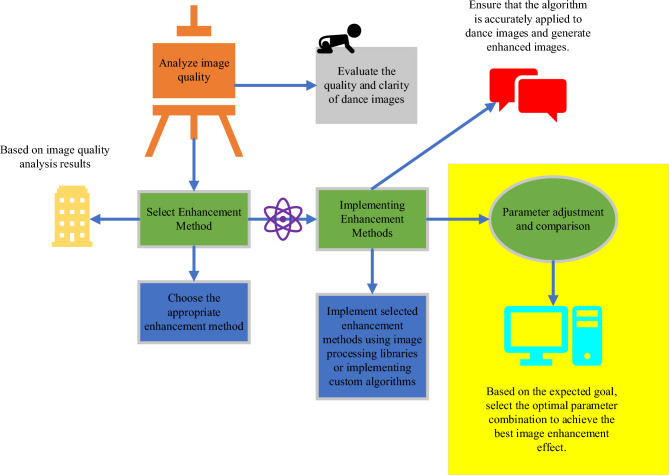
Image preprocessing process.

In folk dance image recognition, image preprocessing and feature extraction play a crucial role in optimizing model design. Firstly, image enhancement improves image quality and clarity, aiding in noise reduction and interference reduction, making dance movements more visibly distinct^23^. Secondly, color space conversion transforms dance images from the RGB color space to other color spaces, extracting richer color features. Choosing an appropriate color space can enhance feature distinctiveness and information content, strengthening the model’s ability to recognize dance movements^24^. Depending on specific circumstances and task requirements, experiments and adjustments should be conducted to determine the color space most suitable for a particular application. Thirdly, the feature extraction network, where convolutional layers in DNN are typically used to extract local features from images^25^. Optimizing the structure and parameter settings of the feature extraction network can enhance its ability to extract key points, contours, and textures in dance movements^26^. Lastly, data augmentation is employed to improve the model’s robustness and generalization ability by using data augmentation techniques to expand the dance image dataset. The image preprocessing optimization method chosen in this study is an image enhancement, with the specific process outlined in Fig. 1.

**Figure 2:**
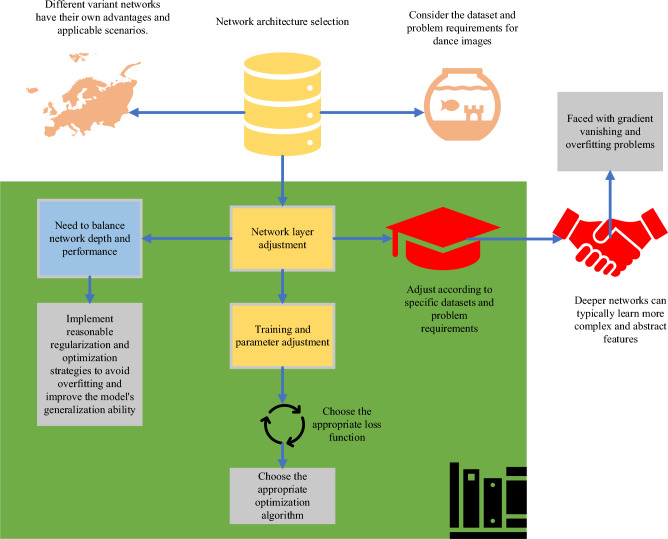
The optimization process of feature extraction.

Image enhancement primarily employs grayscale conversion, transforming a color image into a grayscale image, retaining only brightness information while removing color information. In a grayscale image, each pixel’s value represents the brightness level of that pixel, disregarding its specific color. The main purpose of grayscale conversion is to reduce image complexity, making image details more prominent. Color information in color images may cause visual interference. However, by converting them into grayscale, the focus can be directed toward the brightness and intensity variations in the image, allowing for better analysis and processing of the image. In terms of feature extraction optimization, this study opts for a feature extraction network, a key component of DL, used to extract representative and distinctive feature representations from raw image data. The specific process is illustrated in Fig. 2.

The performance and accuracy of dance image feature extraction can be improved by optimizing the design and tuning of the feature extraction network and using data enhancement technology rationally. When selecting and implementing these optimization methods, it is necessary to consider the dataset’s characteristics, the problem’s requirements, and the training resources’ limitations. Moreover, the training and tuning of the feature extraction network should be carefully analyzed and evaluated to find the best parameter combination and model performance. While end-to-end DL models perform well in many applications, direct application of these models may not yield optimal results in specific application scenarios, such as complex folk dance movement recognition and hand positioning. By combining DL with specific optimization strategies, such as feature pyramid and advanced semantic feature extraction, to improve model performance, this approach meets the specific needs better than a single end-to-end model.

The input of the folk dance training image model based on DNN is usually dance training image data, which is input into the feature extraction network after pretreatment and standardization. Feature extraction network is a part of DNN model, its main task is to extract meaningful and distinguishable feature representation from the original image data. The feature extraction network extracts more abstract feature representations layer by layer through operations such as multi-layer convolution, activation, pooling, and fully connected (FC). Eventually, these features can be passed on to subsequent layers, such as classifiers or regressors, for tasks such as dance movement recognition, pose analysis, and learning recommendations. The convolutional layer plays an important role in the feature extraction network. The local spatial features are extracted by sliding a series of convolution kernels on the input image, and the convolution operation generates the feature map. Convolution kernels of different sizes and numbers can capture features of different scales and semantics. Activation functions, such as ReLU, are generally introduced after each convolutional layer to add nonlinear transformations to extracted features and increase the expressiveness of the network. The pooling layer subsamples the feature map output by the convolutional layer through a sliding window, reduces the size of the feature map, and retains the main feature information. Common pooling operations include maximum pooling and average pooling. The batch normalization layer is used to accelerate the training process and improve the convergence of the model. Normalizing the data makes the network more stable to small changes in the input and facilitates the network to learn more effective features. The FC layer flattens the output of the previous convolutional and pooling layers and connects to the FC layer. The neurons of the FC layer can perform complex nonlinear mapping and provide higher-level semantic feature representation. For the problem of motion blur, the hand in the blurry image is identified by enhancing the feature extraction network. By training the model to recognize hand features under different motion blur conditions, the model’s tolerance to motion blur can be improved.

### Construction of classification model and target detection model in folk dance image recognition

**Figure 3:**
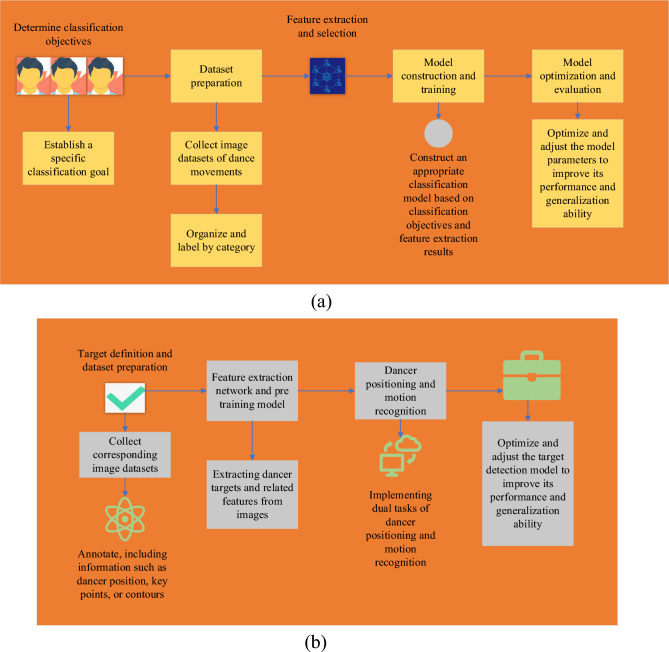
Classification model and target detection model. (**a**) Classification model; (**b**) target detection model.

In folk dance image recognition, the classification and target detection models are common methods for movement recognition and dancer positioning. The model is optimized by optimizing the process of image preprocessing and feature extraction. The optimized classification model and target detection model are presented in Fig. 3.

Figure 3 signifies that in the structure of the classification model, the input layer receives the image data before processing it as input. Feature extraction networks extract high-level, semantically rich feature representations from images. The flatten layer flattens the output of the feature extraction network into a one-dimensional vector. The FC layer generates the final feature representation through nonlinear mapping and feature fusion of multiple FC layers. The output layer is classified by Softmax function according to task requirements. In the structure of the target detection model, the image data before the input layer receives processing is taken as input, the feature extraction network is utilized to extract low-level features and semantic features in the image, and the feature pyramid network is used to process the objects of different sizes. The multi-scale feature pyramid is introduced into the network to realize the target detection of different sizes. The detection layer outputs the position and category information of the target detection box. By optimizing the construction method of the classification model, feature extraction network, target detection model, and training technology, the ability of movement recognition and dancer positioning in folk dance images can be improved. Selecting suitable feature extraction network structures, adjusting model parameters, and optimizing algorithms can improve the model’s performance and accuracy in classification and target detection tasks. At the same time, the quality and quantity of datasets are also essential factors for model optimization, and it is necessary to ensure the adequate and rational use of datasets to improve the model’s generalization ability and robustness. The optimized model relies not only on skin color detection for hand segmentation but also integrates DL feature extraction methods. Employing an advanced semantic feature extraction network can help recognize and differentiate hands from other skin-colored objects. This approach, by learning complex features such as hand shape, size, and contours, enhances segmentation accuracy and robustness beyond mere color reliance. To address the target detection of different sizes, a feature pyramid network is utilized. This structure can handle hand images of varying sizes, improving the model’s recognition capabilities across multiple scales. This is particularly beneficial for distinguishing hands from other similarly colored skin objects as it considers not only color but also shape and size.

When compared to traditional hand segmentation models, the optimization approach proposed in this study provides higher accuracy and robustness under complex conditions. In contrast to traditional skin color detection methods, the proposed optimization approach better handles the diversity and uncertainty encountered in practical applications. Comparisons with traditional convolutional neural network models reveal that the optimized model, with its enhanced feature extraction network, can capture richer semantic information, crucial for recognizing complex actions and postures.

### Experimental design

The experiment selected the University of Florida 101 Human Actions dataset (UCF101), a widely used video action recognition dataset containing 101 categories of action videos, including folk dance. It contains 13,320 video clips covering 101 different categories of actions, including folk dance, sports, daily activities, and more. Each category contains approximately 100–800 samples and is created and maintained by the Computer Vision Laboratory at the University of Central Florida, where each video clip is represented as a sequence of frames with corresponding annotation information. The video frame size and frame rate can be adjusted as needed. At the same time, it is divided into three parts, encompassing the training, verification, and test sets. A common way to divide the sample is to use about 70% of the sample as the training set, about 10% as the validation set, and about 20% as the test set. The video files are stored in Audio Video Interleaved (AVI) or Moving Pictures Experts (MPG) format, and the annotation information is provided in the form of text files and can be downloaded via http://crcv.ucf.edu/data/UCF101.php. The hardware and software configuration parameters required for the experiment are detailed in Table 3.

**Table 3:** Configuration of experimental hardware and software.

Device type	Configuration
Processor	Inter(R) Xeon(R) CPU E5-2620 v4 @ 2.10 GHz
Graphics processing unit (GPU)	NVIDIA Titan Xp 12 GB
Memory	128 GB
Operating system	Ubuntu 16.04 LTS
Programming language	Python 3.6
Technical framework	Py Torch 1.7.0

Part of the code used to run the model in the experiment is as follows:
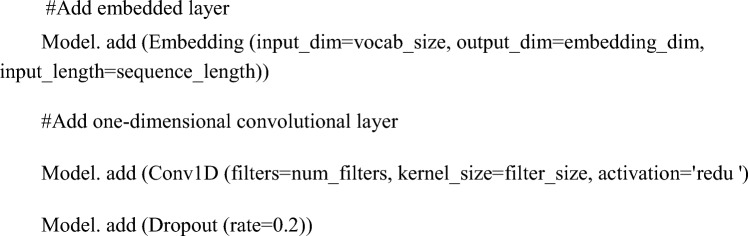


Parameters need to be unified in the experiment, where the number of hidden layer nodes is 8, the learning rate is 0.01, the regularization parameter is 8, the batch size is 8, and the activation function is Relu.

The comparative models chosen in the experiment include the Naive Bayes classifier, decision tree classifier, Logistic Regression (LR), K-Nearest Neighbors (KNN), and Support Vector Machine (SVM). The Naive Bayes classifier is a simple and efficient classification algorithm based on Bayes’ theorem and the assumption of feature conditional independence. It is fast in computation and training, making it suitable for large datasets. Decision trees generate a tree-like structure by partitioning data based on different feature values, providing an intuitive and easily understandable model with good interpretability and visualization. KNN is an instance-based classification algorithm that classifies samples based on the labels of the nearest neighbors in the training set. It is suitable for small datasets and can adapt flexibly to different data distributions. SVM is a well-performing supervised learning algorithm in binary and multiclass tasks, constructing an optimal hyperplane for classification and handling high-dimensional feature spaces and nonlinear data. LR is a commonly used classification algorithm, primarily for binary problems but extendable to multi-class problems. It maps inputs to probability values between 0 and 1 using the sigmoid function and makes classification decisions based on a threshold. One of the reasons for selecting LR as a comparative model is its classical and straightforward nature, making it easier to understand the relationship between model performance and complexity compared to other models. The comparison involves aspects such as body dynamics, dance types, action complexity, movement speed, dance styles, and rhythm. Evaluation indicators encompass classification accuracy, precision, recall, and F1 score. Classification accuracy is a common indicator for assessing the performance of classification models, representing the proportion of correctly classified samples. Precision measures the proportion of true positives among the samples predicted as positive, indicating the accuracy of predicting true positives and evaluating the model’s performance on positives. Recall measures the proportion of true positives among the actual positives, illustrating the model’s ability to find true positives and evaluating its detection performance on positives. F1 score is a combination of precision and recall, used to comprehensively evaluate a classification model’s performance, serving as the harmonic mean of precision and recall.

## Performance analysis of the DNN-based folk dance training image analysis model

### Analysis of horizontal comparison experiment results of folk dance training image classification model

**Figure 4:**
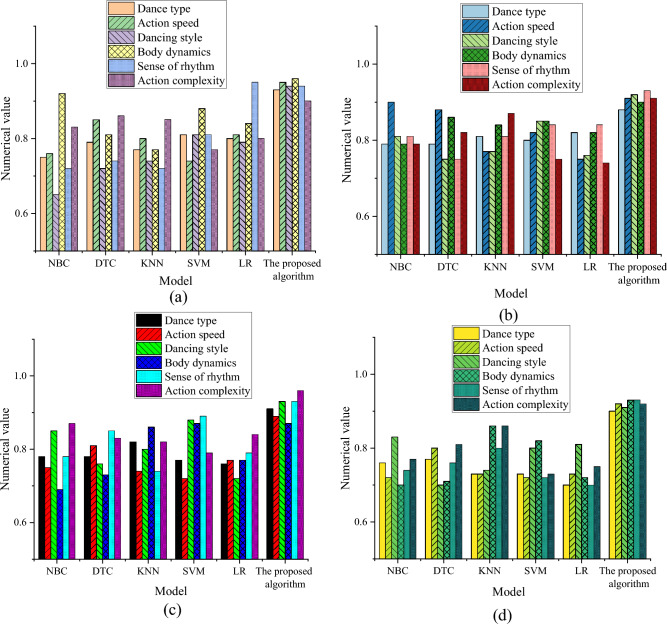
Horizontal comparison experiment results. (**a**) Classification accuracy; (**b**) precision; (**c**) recall; (**d**) F1 value.

The experimental comparison results of the model performance are depicted in Fig. 4.

The results of Fig. 4 signify that the classification accuracy of the optimized model reaches 0.89, 0.93, 0.95, 0.94, 0.96, and 0.94 in terms of movement complexity, dance type, dance style, rhythm, movement speed, and body dynamics. Besides, the optimized model’s performance is much higher than the traditional model’s. These results illustrate that the optimized model has achieved remarkable improvement in classification performance in all aspects. Specifically, compared to the traditional model, the optimized model completed approximately 15.1, 12.3, 12.2, and 18.1% improvements in accuracy, precision, recall, and F1 value, respectively. These results further demonstrate the excellent performance of the optimized model on multiple evaluation indicators.

### Analysis of recognition performance of the target detection model for folk dance training images

**Figure 5:**
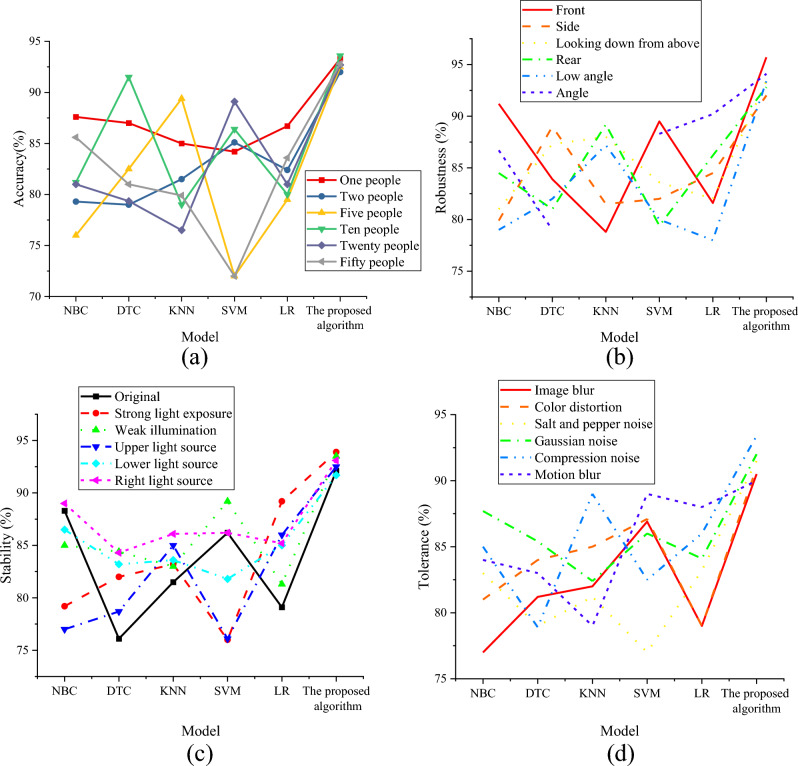
Experimental results of the target detection model. (**a**) Comparison of recognition accuracy of different training images; (**b**) comparison of model robustness with changing perspective; (**c**) comparison of model stability with different lighting conditions; (**d**) comparison of model tolerance under noise interference.

To verify the performance of the optimized target detection model, the experiment researched four influencing factors, including change in perspective, different training images, noise interference, and lighting conditions. The experimental results are suggested in Fig. 5.

According to the results in Fig. 5, it can be observed that the optimized target detection model shows significantly improved recognition performance in folk dance training image classification. In experiments with different numbers of training images, the optimized model presents a high recognition accuracy of more than 92%. This means that even with very limited training data, the model can accurately identify folk dance movements, showing robustness to the amount of data. In the experiment of changing perspective, the optimized model has high robustness, and the recognition accuracy of the model can reach more than 92%, whether it is from a positive perspective or other perspective changes. This shows the model has a stable recognition ability when facing folk dance images from various perspectives. The optimized model reveals excellent stability in the light condition change experiment, with the highest recognition accuracy reaching 93.9%. This indicates that the model can adapt to the changes in different light intensities and directions and still maintain high accuracy for folk dance scenes with complex lighting conditions. In the anti-noise experiment, the optimized model shows above 90% anti-noise capability by introducing noise disturbances such as image blur, color distortion, salt and pepper noise, Gaussian noise, compression noise, and motion blur. This illustrates that in practical applications, even if there is noise interference in folk dance images, the model can still accurately identify dance movements and has good robustness. In summary, this study’s optimized target detection model performs well in folk dance training image classification, with high accuracy, strong robustness, and anti-interference ability. These results offer a vital basis for analyzing folk dance training images and optimizing teaching strategies and provide powerful tools and guidance for researchers, educators, and dance lovers.

### Optimization of folk dance teaching strategy

The optimization of the folk dance teaching strategy is crucial to meet the learning characteristics and demands of students. Learners’ characteristics and needs in folk dance training are vital to formulating optimal teaching strategies. To better meet the requirements of learners, it is necessary to have an in-depth understanding of their physical fitness, skill level, hobbies, learning style, and goals. There are differences in the physical development and flexibility of learners at diverse ages, affecting their performance and learning in dance training. Younger learners may require more basic movement exercises and flexibility training, while adult learners may focus more on technique and artistic expression. Some learners may be beginners unfamiliar with the basic dance movements and combinations and need to build a solid foundation. Some experienced learners may have higher requirements for specific dance styles or techniques that require further challenge and improvement. In addition, it is vital to understand the learner’s interests and learning styles. Various learners may have different interests and preferences in dance, so the teaching content and methods should be designed according to their needs. Simultaneously, some learners prefer visual, auditory, or motor learning styles, which can be combined with multimedia resources and interactive teaching to satisfy the demands of different learning styles.

To optimize the teaching strategy, the design of a personalized learning path is crucial. By making a personalized learning plan according to the characteristics and needs of learners, learning results can be improved, and personal learning goals can be met. Personalized learning path design can also include customized courses and training programs tailored to the needs of learners. For example, some learners may need more repetitive practice and technical reinforcement of basic movements, while others may want to improve stage performance and need more opportunities to participate in performances and presentations. To meet the needs of diverse learners, teachers can flexibly adjust the course arrangement, and provide individual guidance or group training. With the help of an intelligent teaching system, the optimization of folk dance training teaching strategy can be better supported. The intelligent teaching system can automatically adjust the learning content and difficulty according to the learners’ characteristics, needs, and progress, and offer personalized guidance and feedback. Key movement features can be extracted and evaluated by inputting folk dance training images into DNN models. The model can give immediate feedback based on the learner’s action performance, guide the learner to correct and improve, and adjust the subsequent teaching content according to their progress. Immediate feedback and evaluation mechanism is a key component of teaching strategy optimization. By providing timely and accurate feedback and assessment results, learners can quickly understand their performance, identify problems, and correct them. This is conducive to improving learning effectiveness and strengthening self-awareness.

Analyzing learners’ characteristics and needs, designing a personalized learning path, constructing an intelligent teaching system, and analyzing the real-time feedback and evaluation mechanism can optimize the analysis of folk dance training images and teaching strategies by the DNN. This integrated applied approach allows teachers to understand learners better and provide individualized guidance and support based on their needs and characteristics.

## Discussion

It can be seen that the optimized model has significantly improved in the four indicators. Compared with traditional models, the accuracy, precision, recall, and F1 values have increased by approximately 15.1, 12.3, 12.2, and 18.1%, respectively. These results indicate that compared to traditional models, the optimized model performs better in identifying different dance types and movements, promoting the accuracy and stability of classification. In the optimized target detection model, the model’s recognition accuracy is superior to 92% in different training images, illustrating that the optimized model can maintain good performance regardless of whether the training data is small or large. This is very meaningful for applications that require target detection under limited resources. It is particularly noteworthy that the model can still achieve high accuracy even with fewer training images. The optimized model’s robustness in the experiment of changing perspective overtops 92%, which proves that the optimized model has prominent adaptability to various perspective changes, and reaches 95.7% in positive perspectives. In the experiment of changing lighting conditions, the highest stability achieves 93.9%. By introducing changes in different lighting directions and intensities, the robustness of the model is verified. The optimized model can maintain high recognition accuracy under strong and weak lighting conditions. This indicates that the optimized model can effectively adapt to changes in lighting conditions. In the anti-noise test, the model’s tolerance for interference also exceeds 90%, verifying the notable improvement in the recognition performance of the optimized model. The results reveal that the model can maintain relatively stable and high accuracy recognition performance when facing different levels of noise interference.

## Conclusion

As technology and lifestyle continue to advance, folk dance, as a crucial cultural expression, is gaining increasing attention. Therefore, this study optimizes the image analysis model for folk dance training using DNNs. On one hand, the image preprocessing and feature extraction of the DNN are optimized. On the other hand, a classification model and target detection model are established based on the optimized DNN for analyzing folk dance training images. Experimental results reveal that the optimized classification model has improved accuracy in aspects such as dance styles, movement speed, dance types, body dynamics, rhythm, and action complexity, reaching 0.89, 0.93, 0.95, 0.94, 0.96, and 0.94, respectively. The precision values are 0.92, 0.89, 0.90, 0.91, 0.92, and 0.92, while recall values are 0.95, 0.92, 0.88, 0.92, 0.88, and 0.92. F1 scores are 0.90, 0.91, 0.96, 0.92, 0.92, and 0.93, respectively. In summary, the optimized model markedly enhances these four indicators. Compared to traditional models, the optimized model performs better in recognizing diverse dances and movements, improving classification accuracy and stability. Additionally, this study experimentally investigates four influencing factors on the optimized object detection model: noise interference, different training images, changes in perspective, and lighting conditions. The results show that under various training images, the recognition accuracy of the optimized model exceeds 92%. Regarding changes in perspective, the model’s robustness also surpasses 92%, reaching 95.7% for a frontal view. For variations in lighting conditions, the model’s stability reaches up to 93.9%. In the noise resistance experiment, the model’s tolerance to interference also exceeds 90%. These results validate the significant improvement in the optimized model’s recognition performance. This study affords scientific analysis tools and methods for dance teaching and training, facilitating coaches and learners in more effectively correcting and improving dance movements, thus enhancing the quality of teaching and training outcomes.

Although this study has made some achievements, it also has some shortcomings. On the one hand, it mainly focuses on the overall movement recognition of folk dance, and does not involve the fine-grained classification of different dance movements. Future research could further explore how to classify and identify various folk dance movements in detail to better meet the needs of dancers, educators, and researchers. On the other hand, it primarily involves offline image classification analysis. Future research may consider applying the model to real-time classification and interactive systems, which can help provide real-time feedback and guidance, and help dance learners and coaches to better train and demonstrate.

### Supplementary Information

Supplementary Information.

## Data Availability

All data generated or analysed during this study are included in this published article [and its supplementary information files].
