# Supplementary material for: Image analysis and teaching strategy optimization of folk dance training based on the deep neural network
Source: Sci Rep. 2024 May 13;14:10909. doi: 10.1038/s41598-024-61134-y (PMC11091159; doi:10.1038/s41598-024-61134-y)
Supplement: Supplementary file 1 — Supplementary Information. [file 41598_2024_61134_MOESM1_ESM.zip › Code Description.docx]

1. The dataset path (data_dir) and category name (class_names) are specified.

Next, the data is loaded and preprocessed through load_and_preprocess_data functions. This function receives the dataset path and class name as input parameters and returns the divided training and testing sets.

Inside the load_and_preprocess_data function, the code is processed and prepared by following these steps:

Empty lists image_data and labels are created to store image data and corresponding labels.

Each category name is looped through: the category path class_dir is built, concatenating the dataset path with the current category name.

Under the category directory, each file name is traversed: if the file name ends with ".jpg" or ".png" (only image files in the specified format are processed): the image path image_path is established, which is a concatenation of the category path with the current file name.

cv2.imread is used to read images.

The image dimensions are resized to a predefined image_width and image_height.

The image is preprocessed to adjust the pixel value range between [0, 1].

The preprocessed image is added to the image_data list.

The corresponding category name is added to the labels list.

LabelEncoder is adopted to encode labels in the label list, mapping category names to numeric categories (0, 1, 2...）.

to_categorical is used to convert the label list labels into a vector in the form of one-hot encoding.

train_test_split function divides image data and labels into training and test sets, where the test set size accounts for 20% (which can be adjusted according to needs).

To return the divided training set image data (train_images), test set image data (test_images), training set labels (train_labels), and test set labels (test_labels).

Finally, the code calls the load_and_preprocess_data function using the example section and prints out the size of the training and test sets and the number of classes. Then the required modules and classes are imported, including tensorflow, Sequential, Conv2D, MaxPooling2D, Flatten, Dense, and Dropout.

Next, it is necessary to define a function called create_cnn_model that creates a CNN model. This function accepts two parameters: input_shape represents the input image's size and number of channels, and num_classes refers to the number of categories. The following operations are performed inside the function:

An empty sequential model object, model, is created.

By calling the model.add() method, the convolutional layer, the maximum pooling layer, the fully connected layer, and the output layer are added in turn. Specifically, the code adds three convolutional layers through the Conv2D class, each with its input size, convolution kernel size, and activation function (ReLU). Then, three maximum pooling layers are added via the MaxPooling2D class to reduce the spatial dimension of the feature map. Finally, the feature map output by the convolutional layer is flattened into a one-dimensional vector by the Flatten class to enter the fully connected layer.

Before the fully connected layer, a Dense layer containing 128 neurons is added and nonlinearity is introduced using the ReLU activation function.

To reduce the risk of overfitting, a Dropout layer is added after the fully connected layer to inactivate a portion of the connection randomly.

Finally, the output layer defines the number of output neurons based on the number of categories, and the classification probability is output using the Softmax activation function.

After completing the above operations, return the created model object model.

Next, the input image's size and number of channels (input_shape) and the number of categories (num_classes) are specified.

create_cnn_model function creates a CNN model, passing in parameters input_shape and num_classes.

The model.compile method is utilized to compile the model. Here, the optimizer (ADAM), loss function (cross-entropy), and evaluation metric (accuracy) are specified.

Finally, the model.summary() method is called to print the summary information of the model. The summary details important information, such as the name of each model layer, the output shape, and the number of trainable parameters.

The necessary libraries and modules are again imported, including 'cv2' (OpenCV) and 'numpy' (for processing image and array operations), as well as the deep neural network model stored in the 'model.h5' file.

2. An input image shape variable 'input_shape' is defined that corresponds to the model structure.

3. A function called 'preprocess_image' is implemented to preprocess the input frames. This function does the following:

- The input frame is resized to the model’s expected input size.

- The color space of the frame is converted from BGR to RGB.

- Frames are converted to float, and pixel values are normalized between 0 and 1.

- A dimension is added to match the input requirements of the model.

4. A function called 'recognize_action' is implemented to recognize actions using a pre-trained deep neural network model when given a frame of image. This function does the following:

- The 'preprocess_image' function is called to preprocess the input frame.

- The model is used to predict the preprocessed frame and get the prediction result of the action category.

- The 'np.argmax' method obtains the category index with the highest probability in the predicted outcome.

5. A function called 'evaluate_action' is implemented to evaluate key factors such as accuracy of movements, correct posture, and sense of rhythm against preset criteria or guidelines. This function does the following:

- Whether the predicted result is correct based on the predicted action category index and the actual action label (ground_truth).

- Returns the action evaluation result, simply setting the accuracy to 1.0 (prediction correct) or 0.0 (prediction error).

6. In the subsequent part of the code, 'cv2. VideoCapture' is used to turn on the camera and perform a loop to read the video frames continuously.

7. For each frame:

- The 'recognize_action' function is called to identify the action of the image frame and obtain the prediction result of the action category.

- Real action tabs (ground_truth) are simulated (according to the mission set).

- the 'evaluate_action' function is called to evaluate the accuracy of the action.

- Predicted action category index and accuracy are printed.

- 'cv2.imshow' displays the current image frame in the window.

- Wait for the q key to be pressed, and exit the loop if the q key is pressed.

8. The video capture object is released, and the display window is closed.

9. A dictionary of learners' personal information and interests is defined, including name, skill level (skill_level), and interests.

10. A nested dictionary of learning resources corresponding to different skill levels is defined, where each skill level corresponds to a dictionary, which in turn contains a list of learning resources corresponding to different interests and hobbies.

11. A function called 'generate_personalized_learning_path' is implemented to generate personalized learning paths. This function does the following:

- Acquire information from learners about skill levels and interests.

- An empty learning path list is created.

- The learner's hobby list is traversed, and for each hobby, it is determined whether it exists in the learning resource dictionary for the corresponding skill level.

- If present, a list of learning resources corresponding to hobbies is added to the list of learning paths.

- Returns a personalized list of learning paths.

12. The 'generate_personalized_learning_path' function is called, passing in the learner's personal information and learning resources, and saving the generated personalized learning path in the 'personalized_path' variable.

13. Learning paths can be personalized using circular printing. A list of personalized learning paths is traversed and printed as a hyphen for each learning resource.

14. The necessary libraries and modules, including 'cv2' (OpenCV) and 'numpy' (for handling image and array operations) are imported, and the action recognition model is stored in the 'model.h5' file.

15. An input image shape variable 'input_shape' is defined that corresponds to the model structure.

16. A function called 'preprocess_image' is implemented to preprocess the input frame. This function completes the previous omissions to match the 'preprocess_image' function in the previous example.

17. A function called 'recognize_action' is realized to use a pre-trained action recognition model for action recognition when given a frame of the image. This function completes the previous omissions to match the 'recognize_action' function in the previous example.

18. A function called 'evaluate_action' is implemented to evaluate the accuracy of actions according to preset standards or guidelines. This function completes the previous omissions to match the 'evaluate_action' function in the previous example.

19. An indicator function 'calculate_difference' for calculating variance or error is defined, which defines the method for calculating variance or error according to actual needs and returns the value of the difference or error.

20. A function 'is_successful' defines a threshold to judge success or not, judging success based on the difference or error and threshold, returning 'True' if the difference or error is less than the threshold, otherwise returning 'False'.

21. A function called 'process_frame' is implemented to process video frames and provide feedback. This function does the following:

- The input frame is preprocessed.

- The 'recognize_action' function is utilized to identify the preprocessed frame's action and obtain the action category's prediction result.

- The 'evaluate_action' function is used to evaluate the accuracy of the action.

- The 'calculate_difference' function calculates the difference or error between the predicted result and the real action label.

- The 'is_successful' function determines success based on differences, errors, and thresholds.

- Further feedback and evaluation based on the results are carried out, such as displaying text on image frames and adjusting colors.

- Returns the processed image frame.

22. In the rest of the code, use 'cv2. VideoCapture' turns on the camera and performs a loop to read the video frames continuously.

23. For each frame:

- the 'process_frame' function is called to process and feedback on the image frame, passing in the real action label and difference threshold.

- 'cv2.imshow' is employed to display the processed image frame.

- Wait for the q key to be pressed, and exit the loop if the q key is pressed.

24. Ultimately, the video capture object is released, and the display window is closed.
